# Clinical Outcomes of Diabetic Ketoacidosis in Type 2 Diabetes Patients with and without SGLT2 Inhibitor Treatment: A Retrospective Study

**DOI:** 10.3390/biomedicines11102689

**Published:** 2023-10-01

**Authors:** Afif Nakhleh, Areen Othman, Amin Masri, Moshe Zloczower, Sagit Zolotov, Naim Shehadeh

**Affiliations:** 1 Institute of Endocrinology, Diabetes and Metabolism, Rambam Health Care Campus, Haifa 3109601, Israel; 2 Diabetes and Endocrinology Clinic, Maccabi Healthcare Services, Haifa 3299001, Israel; 3 The Azrieli Faculty of Medicine, Bar-Ilan University, Safed 1311502, Israel; 4 Ruth & Bruce Rappaport Faculty of Medicine, Technion, Israel Institute of Technology, Haifa 3525433, Israel

**Keywords:** type 2 diabetes mellitus, diabetic ketoacidosis, SGLT2 inhibitors, mortality from any cause

## Abstract

Aim: This study aimed to compare the clinical course and outcomes of DKA in T2DM patients who received treatment with SGLT2 inhibitors versus those who did not. Methods: A retrospective analysis was conducted on T2DM patients who were admitted to the Rambam Health Care Campus with DKA between 7/2015 and 9/2020. Demographic, clinical, and laboratory data were obtained from electronic medical records. Outpatient mortality was monitored until 12/2022. Results: Of 71 T2DM patients admitted with DKA, 16 (22.5%) were on SGLT2 inhibitor treatment upon admission. SGLT2 inhibitor users had a higher BMI and were less likely to be treated with insulin. During hospitalization, the rates of acute kidney injury, concomitant infections, and inpatient mortality among SGLT2 inhibitor users were comparable to non-users. The median follow-up period was 35.1 months for the SGLT2 inhibitor users and 36.7 months for non-users. The long-term mortality from any cause was lower among the SGLT2 inhibitor users (12.5% vs. 52.7%, *p* = 0.004). In Cox regression analysis, SGLT2 inhibitor use was associated with a lower risk of long-term mortality from any cause (HR = 0.19, *p* = 0.04). Conclusion: T2DM patients with DKA who received SGLT2 inhibitors had lower long-term mortality from any cause compared to those who did not receive SGLT2 inhibitors.

## 1. Introduction

Diabetic ketoacidosis (DKA) is a serious complication that is associated with an increased risk of outpatient mortality in patients with diabetes [1]. Indeed, the modern management of DKA has reduced mortality rates among hospitalized patients. Nevertheless, despite significant advancements in inpatient care, individuals who experience an episode of DKA are at higher risk of outpatient mortality, particularly those with type 2 diabetes (T2DM), with a mortality rate as high as 30% within one year [2].

Sodium-glucose cotransporter 2 (SGLT2) inhibitors lower the plasma glucose concentration by inhibiting the tubular SGLT2 transporter and causing glycosuria. Multiple studies have demonstrated several benefits of SGLT2 inhibitors on cardiovascular and renal outcomes in patients with and without type 2 diabetes [3,4,5,6]. The existing evidence derived from randomized controlled and observational studies of SGLT2 inhibitors indicates a minimal risk of DKA in individuals with T2DM [7]. Based on a systemic review and meta-analysis of 39 randomized controlled trials involving 60,580 patients, there is strong evidence indicating that SGLT2 inhibitors can increase the risk of DKA in individuals with T2DM compared to other antidiabetic drugs or placebos. The relative risk is 2.13, and the absolute rate is three events per 1000 patient-years [8].

The occurrence of SGLT2-inhibitor-associated DKA does not seem to be limited to any specific demographic or comorbid subpopulations, and it can manifest at any point during the duration of SGLT2 inhibitor use [9]. Therefore, understanding the clinical course of DKA in patients with T2DM receiving SGLT2 inhibitors is essential. 

The objectives of the study were to compare the clinical characteristics and short- and long-term mortality outcomes after DKA in T2DM patients who received treatment with SGLT2 inhibitors versus those who did not.

## 2. Materials and Methods

This retrospective cohort study was conducted using the electronic medical database of the Rambam Health Care Campus (RHCC), a tertiary hospital in Northern Israel. We retrieved data on adult patients with T2DM who were admitted with DKA between July 2015 and September 2020. The RHCC institutional review board (IRB) and ethics committee approved accessing and analyzing data. Due to the anonymized nature of patient records, individual patient informed consent was not required.

T2DM was diagnosed using a comprehensive approach that included examining electronic health records, utilizing ICD-9 coding for diabetes, analyzing medication usage, reviewing laboratory values (including C-peptide measurements and antibody testing), and examining past outpatient clinic visits. We included individuals who had been diagnosed with T2DM at least three years before the admission with DKA. Those who had received insulin treatment within three years of their diabetes diagnosis were excluded. To reduce the chances of enrolling people with latent autoimmune diabetes of the adult (LADA), individuals under the age of 40 with a BMI less than 30 kg/m^2^ and no family history of diabetes were excluded. The confirmation of DKA diagnosis was based on the fulfillment of the following criteria: an arterial pH level equal to or less than 7.3, a serum bicarbonate level equal to or less than 18 mmol/L, and the presence of ketones in the urine [10]. It is important to note that a blood glucose level exceeding 250 mg/dL was not mandatory for the diagnosis, as some patients might present with euglycemic DKA. The local DKA management protocol was based on the American Diabetes Association guidelines [10].

The index date was set as the date of admission with DKA, while the end of follow-up occurred either at the time of death or by 31 December 2022.

At the index date, we documented the following parameters: (1) anthropometric data including age, gender, body mass index (BMI), and duration of diabetes; (2) details of glucose-lowering therapy used within 48 h before the index date; (3) recent use of systemic glucocorticoids during the week preceding the index date; (4) clinical diagnoses (using ICD-9 clinical diagnostic codes), including arterial hypertension, hyperlipidemia, coronary artery disease, chronic heart failure, stroke, peripheral vascular disease, diabetic neuropathy, diabetic retinopathy, chronic kidney disease (CKD) based on ICD-9 codes and at least one measurement of an estimated glomerular filtration rate (eGFR) < 60 mL/min/1.73 m^2^ within the year before hospital admission, alcohol use, smoking, and drug abuse status; (5) signs on admission including level of consciousness, Kussmaul breathing, and heart rate; and (6) initial blood test results of glucose, HbA1c, creatinine, blood urea nitrogen (BUN), sodium (Na), potassium (K), chloride (Cl), pH, bicarbonate (HCO3), partial pressure of carbon dioxide (pCO2), anion gap, hemoglobin (Hb), and white blood cell count (WBC). To assess glycemic control over the past year, we examined the average of all HbA1c levels measured 3 to 12 months before admission for each patient.

We examined precipitating factors of DKA, duration of hospital stay (in days), and rates of concurrent infections during hospitalization, as well as the in-hospital mortality and long-term mortality from any cause.

**Figure 1:**
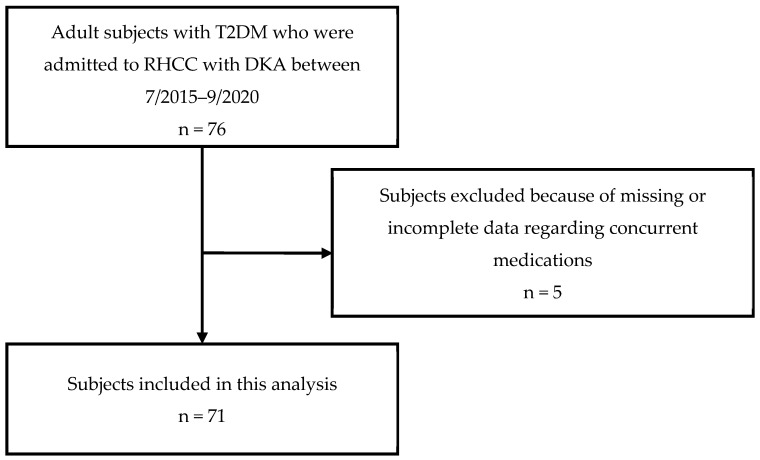
CONSORT diagram of subjects included in the study.

In total, 71 out of 76 T2DM patients with DKA were included in the study (Figure 1).

### Statistical Analysis

Categorical variables were described by frequencies and percentages. Continuous and count variables were described using mean (±standard deviation [SD]). To assess significant differences in clinical characteristics and outcomes between patients who received treatment with SGLT2 inhibitors and those who did not, categorical variables were analyzed using the Fisher exact test, and continuous variables were analyzed using Student’s *t*-test. Kaplan–Meier cumulative survival curves were plotted for patients who received SGLT2 inhibitors and those who did not, and a log-rank test was used to compare the survival rates between the two groups. A Cox regression analysis was conducted to examine the relationship between SGLT2 inhibitor use and long-term mortality from any cause while adjusting for potential confounders that differed between groups (with *p*-value < 0.1). Unless stated otherwise, a two-sided *p*-value < 0.05 was considered statistically significant. All analyses were conducted with the statistical software SPSS, version 25 (IBM Corporation, Armonk, NY, USA).

## 3. Results

Of the 71 T2DM patients admitted with DKA, 16 (22.5%) were on SGLT2 inhibitor treatment upon admission, of whom 10 were using empagliflozin and 6 were using dapagliflozin. Table 1 presents the baseline characteristics of the SGLT2 inhibitor user and non-user groups. There were no significant differences observed in terms of age, gender distribution, and duration of diabetes between the two groups. The mean HbA1c before admission was 10.3 ± 3.6% in the SGLT2 user group and 10.5 ± 2.3% in the non-user group (*p* = 0.79). SGLT2 inhibitor users had a higher BMI (28.8 ± 4.7 vs. 25.8 ± 4.6, *p* = 0.03), were more likely to present with glucose levels below 300 mg/dl (44% vs. 12.7%, *p* = 0.01), and were less likely to be treated with insulin at admission than non-users (37.5% vs. 69.1%, *p* = 0.04) (Table 1). While there were no statistically significant differences in comorbid conditions between the groups, there was a slight trend towards a higher prevalence of hypertension, coronary artery disease, and CKD among non-users of SGLT2 inhibitors. On the other hand, SGLT2 inhibitor users had a numerically higher prevalence of peripheral vascular disease.

The precipitating factors of DKA are listed in Table 2. The most common precipitating factors of DKA were infections, followed by nonadherence to treatment. No DKA episodes were attributed to COVID-19 infection.

The therapeutic approach did not differ between the two groups, with all patients receiving intravenous regular insulin and fluid resuscitation. The average hospital stay was 2.2 ± 2.6 days for SGLT2 inhibitors users and 2.7 ± 2.9 days for the non-users. During hospitalization, the rates of concomitant infections among SGLT2 inhibitor users were comparable to those in the non-user group (7/16 (43.7%) vs. 23/55 (41.8%), *p* = 1, respectively). The inpatient mortality rate was 2/16 (12.5%) among SGLT2 inhibitor users and 11/55 (20%) among non-users (*p* = 0.72).

**Figure 2:**
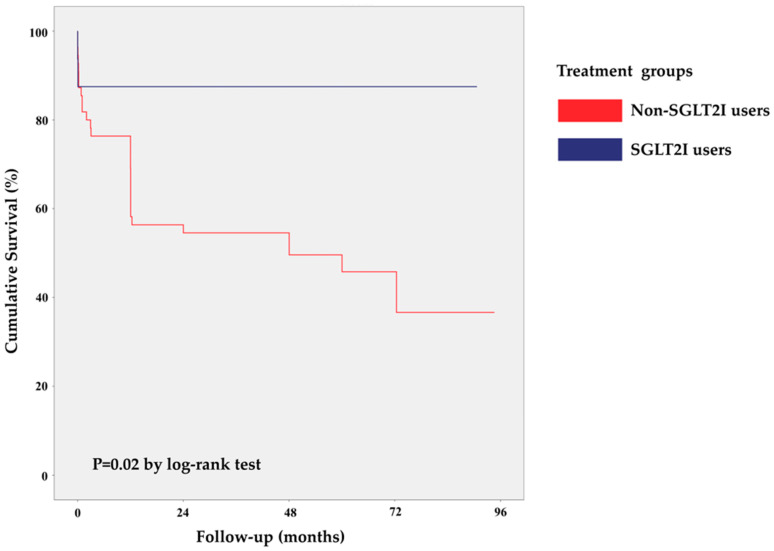
Kaplan–Meier survival curves for SGLT2 inhibitor users and non-users during follow-up.

The median follow-up period for the SGLT2 inhibitor users was 35.1 months and for the non-users was 36.7 months. The long-term mortality from any cause was significantly lower among the SGLT2 inhibitor users (2/16 (12.5%) vs. 29/55 (52.7%), *p* = 0.004), which was further confirmed by the log-rank test demonstrating higher cumulative overall survival in the SGLT2 inhibitor users’ group (*p* = 0.02) (Figure 2). In the Cox regression analysis that adjusted for BMI, insulin use, glucose, BUN, pCO2, and Cl values at admission, SGLT2 inhibitor use was associated with a lower risk of long-term mortality from any cause (HR = 0.19, 95% CI [0.04–0.90], *p* = 0.04). 

Table 3 highlights the causes of mortality during follow-up, with infections as the predominant cause.

## 4. Discussion

The present study showed that 22.5% of T2DM patients admitted with DKA were on SGLT2 inhibitor treatment. SGLT2 inhibitor users had a higher BMI and were less likely to be treated with insulin at admission compared to non-users. The study observed comparable rates of concomitant infections and inpatient mortality between SGLT2 inhibitor users and non-users. However, SGLT2 inhibitor users had significantly lower long-term mortality from any cause compared to those who did not receive SGLT2 inhibitors (12.5% vs. 52.7%, *p* = 0.004) during a median follow-up period of ~3 years. 

Indeed, several studies have demonstrated an increased outpatient mortality risk following an episode of DKA. In a study of 284 patients (including 87 with T2DM), the observed mortality rate among individuals with T2DM after one year of follow-up was 30%, and, in line with our findings, most deaths were attributed to infections. In that study, factors associated with an increased risk of mortality within one year following hospital discharge included advanced age, vascular complications related to diabetes, cognitive impairment, and residing in a care facility [2]. In another study conducted in a Scottish intensive care unit comprising 386 individuals, the mortality rate within one year following DKA was 18%, and it increased to 35% at the end of five years [11]. Additionally, a Taiwanese cohort study involving 4517 elderly participants aged 65 years or older, who experienced hyperglycemic crises, demonstrated a one-year mortality rate of 19% and a six-year mortality rate of 36% during the subsequent follow-up period [12]. 

The long-term mortality rate among non-users of SGLT2 inhibitors seems to be consistent with the existing literature. In contrast, our study has revealed a considerably lower long-term mortality rate following DKA among users of SGLT2 inhibitors. Furthermore, employing a Cox regression analysis that accounted for variables that differed between groups, the use of SGLT2 inhibitors was associated with a reduced risk of long-term mortality from any cause.

This finding is noteworthy and prompts several potential explanations. The nature of the DKA events in T2DM may differ between SGLT2 inhibitor users and non-users. DKA events observed in SGLT2 inhibitor users may reflect a transient metabolic deterioration that is more responsive to treatment and correction. In contrast, DKA events in the non-users of SGLT2 inhibitors may indicate a progressive metabolic deterioration, potentially reflecting a more severe disease course that leads to increased morbidity and mortality. This is supported by the fact that non-users of SGLT2 inhibitors were more likely to require insulin therapy, which appears to be a marker of more severe insulin deficiency. Progressive insulin deficiency is a recognized risk factor for poor outcomes in T2DM [13]. The presumably higher prevalence of insulin deficiency among non-users of SGLT2 inhibitors suggests a more severe metabolic disturbance, which could have contributed to higher long-term mortality. 

Furthermore, a slightly higher proportion of comorbid conditions, such as hypertension, coronary artery disease, and CKD, was observed in this group. Although the difference was statistically insignificant, it might partially explain the divergent long-term outcomes observed between the two groups.

Both groups had significant hyperglycemia for several months before DKA. Prolonged exposure to elevated glucose concentrations can lead to pancreatic beta cell dysfunction which increases the risk for DKA [14]. However, in cases of SGLT2-inhibitor-induced DKA, it could be hypothesized that there is a relatively milder insulin deficiency, indicating a less severe clinical progression and a more favorable prognosis once the causative factor (SGLT2 inhibitor) is discontinued [14].

On the other hand, the cardiorenal protective effects of SGLT2 inhibitors may have contributed to improved long-term outcomes in patients with DKA. SGLT2 inhibitors have been shown to improve cardiovascular outcomes in patients with T2DM by reducing the risk of major adverse cardiovascular events and heart failure hospitalizations and have demonstrated renoprotective effects by slowing the progression of diabetic kidney disease [3,4,5,6].

In our study, we found that the average hospital stay for patients with DKA was approximately 2.6 days. This contrasts with a previous study in South Korea that reported an average stay of 10.8 days, which is longer than what we observed [15]. Additionally, a study conducted in the United Arab Emirates (UAE) involving 55 patients with T2DM mellitus admitted with DKA found that SGLT2 inhibitor users were about five times more likely to experience a prolonged hospital stay of ≥14 days compared to non-users [16]. We cannot explain these disparities. However, our findings are consistent with previous reports from Israel, where most patients with DKA had a median hospital stay of 3 days [17].

The UAE study did not find a significant difference in DKA complications and mortality rates between SGLT2 inhibitor users and non-users. However, none of the three patients who died during hospitalization were SGLT2 inhibitor users, and the long-term outcomes after discharge were not compared [16]. Similarly, our study demonstrated comparable inpatient mortality rates between SGLT2 inhibitor users and non-users, but there were significant differences in the long-term prognosis. This underscores the importance of closely monitoring T2DM patients after DKA.

The identification of patients at an increased risk of developing DKA is essential, particularly those with advanced beta cell dysfunction who require insulin therapy and those at higher risk of complications, such as infections. This emphasizes the importance of implementing a multifactorial treatment for patients with T2DM, as it has been demonstrated to improve outcomes in this population [18].

Since the introduction of SGLT2 inhibitors, the medical community has gained a better understanding of euglycemic DKA. While severe infections are the most common cause, there are several factors that can increase the risk of developing this condition. These factors include a long history of type 2 diabetes, poor glycemic control, and coexisting conditions such as alcohol consumption and pancreatic insufficiency [19,20]. The symptoms of euglycemic DKA are similar to those of hyperglycemic DKA, but blood glucose levels are normal, making it difficult to diagnose. However, with early identification and proper management, prognosis is generally good. A recent meta summary of case reports involving 169 patients showed a low mortality rate of 2.4% [20]. 

Physicians treating patients taking SGLT2 inhibitors should be aware of this potential complication and educate their patients for early diagnosis and treatment. The European Medicines Agency recommends the immediate discontinuation of SGLT2 inhibitors if symptoms or signs of DKA are suspected, and not starting them again until DKA is ruled out and any underlying factors have been identified, addressed, and resolved [21].

This study has some limitations, including its retrospective design and the potential for unidentified confounding variables to have influenced our results, limiting our ability to draw causal inferences. While the treatment approach appeared to be similar for both groups, we cannot rule out the possibility that there were differences in the doses of insulin or the types and volumes of fluids used, which may have affected our results. It is important to note that CKD was identified using ICD-9 codes and eGFR measurements only. Albuminuria was not used as a classification criterion for CKD in this study because we could not obtain enough data, before or during hospitalization. This may impact the overall evaluation of CKD status among the patients examined. Furthermore, being a single-center small-cohort study conducted in Northern Israel, the generalizability of our findings may be limited. The comprehensive examination of electronic health records, in conjunction with the rigorous exclusion criteria, makes it unlikely for patients with undiagnosed LADA to be included, although absolute exclusion cannot be definitively ascertained.

More research is required to determine the effects of SGLT2 inhibitors on the clinical course of DKA treatment, given the limited number of DKA cases associated with these inhibitors in our cohort (n = 16) and the differences between our study groups.

In summary, this study provides two key findings. First, it confirms the concerning observation regarding the poor prognosis of DKA in T2DM and highlights the urgent need for the proactive monitoring and meticulous care for patients after an episode of DKA. Second, the study introduces the idea that most T2DM patients who developed DKA while using SGLT2 inhibitors have a relatively favorable long-term prognosis, suggesting that DKA in this group may have a different underlying nature. However, larger studies are needed to validate these findings.

## Figures and Tables

**Table 1:** Baseline characteristics of SGLT2 inhibitor users and non-users.

Characteristic	Non-SGLT2I Users (n = 55)	SGLT2I Users (n = 16)	*p*-Value
Age—yr	67.6 ± 14.4	64.6 ± 11.0	0.44
Female—no. (%)	25 (45.5)	7 (43.8)	1
BMI—kg/m^2^	25.8 ± 4.6	28.8 ± 4.7	0.03
Median duration of T2DM (range)—yr	7.5 (3–17)	6 (4–13)	0.56
HbA1c before admission—%	10.5 ± 2.3	10.3 ± 3.6	0.79
Medical history—no. (%)			
Hypertension	44 (80.0)	10 (62.5)	0.19
Hyperlipidemia	39/53 (73.6)	12 (75)	1
Coronary artery disease	21 (38.2)	4 (25)	0.39
Chronic heart failure	13 (23.6)	3 (18.8)	1
Stroke	8/54 (14.8)	2 (12.5)	1
Peripheral vascular disease	13/54 (24.1)	6 (37.5)	0.34
Chronic kidney disease *	15 (27.3)	2 (12.5)	0.33
Diabetic neuropathy	10/54 (18.5)	3 (23.1)	0.72
Diabetic retinopathy	7/54 (13)	3 (18.8)	0.69
Current smoker	21 (38.2)	5 (31.3)	0.77
Alcohol use disorder	7 (12.7)	1 (6.3)	0.67
Drug abuse	0	0	1
Concurrent medications—no. (%)			
Metformin	22 (40)	9 (56.3)	0.27
Sulfonylurea	6 (10.9)	0	0.33
DPP4 inhibitor	2 (3.6)	1 (6.3)	0.54
GLP-1 receptor agonist	1 (1.8)	1 (6.3)	0.40
Insulin	38 (69.1)	6 (37.5)	0.04
Pioglitazone	2 (3.6)	0	1
Systemic glucocorticoid	3/53 (5.7)	1 (6.3)	1
Signs on admission, no. (%)			
Unconsciousness	8/54 (14.8)	2 (12.5)	1
Kussmaul breathing	20/54 (37)	3 (18.8)	0.23
Heart rate—bpm	102 ± 17	101 ± 19	0.99
Initial blood tests			
Glucose—mg/dL	491 ± 159	385 ± 143	0.02
Glucose < 300mg/dL, no. (%)	7/55 (12.7)	7/16 (44)	0.01
Creatinine—mg/dL	2.3 ± 1.5	1.8 ± 0.9	0.25
Blood urea nitrogen—mg/dL	36.6 ± 27.9	23.9 ± 9.9	0.007
Sodium—mmol/L	132.5 ± 6.2	135.3 ± 6.6	0.16
Potassium—mmol/L	4.9 ± 0.9	4.6 ± 0.7	0.27
Chloride—mmol/L	98.6 ± 6.9	102.1 ± 7	0.09
Osmolality—mOsm/kg	342 ± 44	330 ± 31	0.33
pH	7.14 ± 0.13	7.16 ± 0.10	0.63
Bicarbonate—mmol/L	15.2 ± 7	13.3 ± 3.8	0.15
pCO2—mmHg	33 ± 13.1	43.2 ± 11.5	0.007
Anion gap—mmol/L	19.7 ± 8.5	20.7 ± 6	0.69
Hemoglobin—g/dL	12.7 ± 2.5	13.6 ± 2.2	0.20
White blood cells—×10^−9^/L	14.7 ± 6.9	13.9 ± 4.6	0.65
HbA1c—%	10.1 ± 1.9	10.7 ± 3.4	0.57

Plus-minus values are means ± SD. SGLT2I denotes SGLT2 inhibitor. * Chronic kidney disease was identified using ICD-9 clinical diagnostic codes and at least one measurement of eGFR < 60 mL/min/1.73 m^2^ within the year before hospital admission.

**Table 2:** Precipitating factors of DKA among SGLT2 inhibitor users and non-users.

	Non-SGLT2I Users (n = 55)	SGLT2I Users (n = 16)	*p*-Value
Cause of DKA n, %			0.47
Unknown	9 (16.4)	4 (25.0)	
Nonadherence to treatment *	12 (21.8)	3 (18.8)	
Nonadherence to insulin	7 (58.3)	2 (66.7)	
Infection	27 (49.1)	6 (37.5)	
Urinary tract infection	8 (29.7)	2 (33.3)	
Pneumonia	9 (33.3)	1 (16.7)	
Acute bronchitis	2 (7.4)	0	
Acute gastroenteritis	2 (7.4)	0	
Wound infection	1 (3.7)	1 (16.7)	
Acute diverticulitis	1 (3.7)	0	
Perianal abscess	1 (3.7)	0	
Acute sinusitis	0	1 (16.7)	
Acute pharyngitis	0	1 (16.7)	
Viral myocarditis	1 (3.7)	0	
Unknown origin	2 (7.4)	0	
Acute kidney injury	2 (3.6)	1 (6.3)	
Alcohol use	1 (1.8)	1 (6.3)	
Cardiovascular event	1 (1.8)	0	
Trauma	3 (5.5)	0	
Corticosteroids	0	1 (6.3)	

SGLT2I denotes SGLT2 inhibitor. * Treatment nonadherence was defined as refilling less than 70% of prescribed glucose-lowering medication within the year before hospital admission.

**Table 3:** Causes of mortality during follow-up among SGLT2 inhibitor users and non-users.

	Non-SGLT2I Users (n = 29)	SGLT2I Users (n = 2)	*p*-Value
Cause of mortality n, %			0.92
Unknown	1 (3.4)	0	
Infection	23 (79.3)	2 (100)	
Pneumonia	9 (39.1)	0	
Urinary tract infection	7 (30.5)	1 (50)	
Wound infection	2 (8.7)	1 (50)	
Acute encephalitis	1 (4.3)	0	
Viral myocarditis	1 (4.3)	0	
Unknown origin	3 (13.1)	0	
Acute kidney injury	4 (13.8)	0	
Gastrointestinal bleeding	1 (3.4)	0	

SGLT2I denotes SGLT2 inhibitor.

## Data Availability

The data presented in this study are available upon request from the corresponding author.
